# Metastatic Renal Cell Carcinoma Management: From Molecular Mechanism to Clinical Practice

**DOI:** 10.3389/fonc.2021.657639

**Published:** 2021-04-22

**Authors:** Michela Roberto, Andrea Botticelli, Martina Panebianco, Anna Maria Aschelter, Alain Gelibter, Chiara Ciccarese, Mauro Minelli, Marianna Nuti, Daniele Santini, Andrea Laghi, Silverio Tomao, Paolo Marchetti

**Affiliations:** ^1^ Department of Clinical and Molecular Medicine, Sapienza University of Rome, Rome, Italy; ^2^ Department of Medical-Surgical Sciences and Translational Medicine, Sapienza University of Rome, Rome, Italy; ^3^ Medical Oncology Unit, Policlinico Umberto I, Sapienza University of Rome, Rome, Italy; ^4^ Medical Oncology Unit, Azienda Ospedaliero Universitaria Sant’Andrea, Rome, Italy; ^5^ Department of Medical Oncology, Fondazione Policlinico Universitario Agostino Gemelli IRCCS, Rome, Italy; ^6^ Department of Medical Oncology, Azienda Ospedaliera San Giovanni Addolorata, Rome, Italy; ^7^ Department of Experimental Medicine, University of Rome Sapienza Rome, Rome, Italy; ^8^ Department of Medical Oncology, University Campus Bio-Medico, Rome, Italy; ^9^ Department of Radiological, Oncological and Anatomo-Pathological Sciences, Policlinico Umberto I, Sapienza University of Rome, Rome, Italy

**Keywords:** renal cancer carcinoma, targeted therapy, tyrosine kinase inhibitor (TKI), immune checkpoints inhibitor, new biomarkers

## Abstract

The therapeutic sc"enario of metastatic renal cell cancer (mRCC) has noticeably increased, ranging from the most studied molecular target therapies to those most recently introduced, up to immune checkpoint inhibitors (ICIs). The most recent clinical trials with an ICI-based combination of molecular targeted agents and ICI show how, by restoring an efficient immune response against cancer cells and by establishing an immunological memory, it is possible to obtain not only a better radiological response but also a longer progression-free and overall survival. However, the role of tyrosine kinase inhibitors (TKIs) remains of fundamental importance, especially in patients who, for clinical characteristics, tumor burden and comorbidity, could have greater benefit from the use of TKIs in monotherapy rather than in combination with other therapies. However, to use these novel options in the best possible way, knowledge is required not only of the data from the large clinical trials but also of the biological mechanisms, molecular pathways, immunological mechanisms, and methodological issues related to both new response criteria and endpoints. In this complex scenario, we review the latest results of the latest clinical trials and provide guidance for overcoming the barriers to decision-making to offer a practical approach to the management of mRCC in daily clinical practice. Moreover, based on recent literature, we discuss the most innovative combination strategies that would allow us to achieve the best clinical therapeutic results.

## Introduction

Renal cancer is the 10th most common cancer in Italy, with approximately 13,400 new cases per year (1), 70–80% have clear cell histology, while papillary, medullary, chromophobe, and other forms classified as non-clear cell histology are rare. Approximately 25% of patients present with the advanced-stage disease since their diagnosis, and among those undergoing nephrectomy, about one-third experience a distant recurrence during the rest of their lives and are initiated to systemic treatment.

Despite the significant therapeutic improvements, the 5-year survival rate of patients with metastatic renal cell cancer (mRCC) remains poor, especially in patients with unfavorable prognostic factors (2). The two validated prognostic models for the classification of patients with mRCC within clinical trials are the Memorial Sloan Kettering Cancer Centre (MSKCC) model (3) and the International mRCC Database Consortium (IMDC) that date back to 2005 and 2009, respectively (4). Although more than 10 years have elapsed, and in the meantime, drug molecules with new mechanisms of action have been developed, clinical trials still stratify patients into those with favorable (with 0 poor prognostic factors), intermediate (with 1–2 poor prognostic factors), or poor risk in the presence of at least three of the following prognostic factors: less than 1 year from diagnosis to treatment time, a Karnofsky PS score of <80 at the start of treatment, anemia, neutrophil or platelet count greater than the normal upper limit, or hypercalcemia (corrected Ca >10 mg/dl or >2.5 mmol/L).

The therapeutic scenario of mRCC has undergone incredible enrichment in recent years, ranging from the most studied tyrosine kinase inhibitor (TKI)-targeted therapies (anti-vascular endothelial growth factor (VEGF) and anti-mTOR) to those most recently introduced (anti-MET, anti-RET, and anti-FGFR) up to immunotherapy (IO) (anti-PD-1, anti-PD-L1, and anti-CTLA-4). Literature data on new therapeutic indications with cabozantinib in both the first and second lines (5, 6), nivolumab after anti-VEGF TKI progression (7), nivolumab combined with ipilimumab in naive patients with poor prognostic factors, and pembrolizumab combined with axitinib in all prognostic subgroups (8), have modified the prognosis of patients with mRCC. Especially, patients classified as ‘intermediate’ risk pass from a historical median survival of approximately 20 months to 3 years in the front line, which is almost equal to that of patients with favorable prognosis. On the one hand, we have seen a considerable improvement in the therapeutic algorithm of mRCC [clinical guidelines reported different therapeutic options only in patients with intermediate prognosis (1)]. On the other hand, the rate of development in the identification of new prognostic and predictive factors has not been the same throughout. Therefore, MSKCC/IMDC remains the standard prognostic classification criteria. However, in light of the complex mechanisms of action of the new TKI molecules, such as cabozantinib or combinations of TKIs and IO, or even more combinations of different immune checkpoint inhibitors (ICIs), are we sure that these ‘old’ criteria are sufficient?

In the era of precision medicine, in which knowledge of the molecular and genomic aspects of renal cancer has become ever wider, how can we think that criteria based on obvious clinical considerations (poor performance status and a short progression-free interval) and hematochemical parameters are sufficient to determine a therapeutic choice? To make the best use of new drugs and associations and to propose new therapeutic sequences, better knowledge is required not only of the data derived from the large clinical trials but also of the basic biology, the complexity of involved molecular pathways, the immunology of tumours, and methodological problems related to both new response criteria and new endpoints. In this complex scenario, this review aims to provide a practical approach to the management of advanced renal cancer, framing the new results in daily clinical practice and providing points for reflections to overcome decision-making barriers based on physician therapeutic choice.

## The Heterogeneity of Renal Tumor

RCC includes a heterogeneous group of tumors that are characterized by different clinical and genomic factors and are increasingly well defined in both syndromic and sporadic settings (9). These tumor types originate from different cells; for example, clear cell and papillary carcinomas arise from the proximal or parietal kidney cells, whereas chromophobe carcinomas arise from the intercalated cells (10) and are characterized by different genomic drivers that lead to tumorigenesis. In more than 90% of clear cell RCC cases, large-scale genomic sequencing has identified chromothripsis of chromosome 3p, typically with a concurrent gain of 5q (>67%) and loss of 15q (45%) (9). In particular, the loss of 3p results in the inactivation of Von Hippel–Lindau disease tumor suppressor protein (VHL). Mutations in genes encoding other components of the VHL complex [such as TCEB1 (also known as ELOC)] also lead to VHL inactivation (11–13). pVHL is part of a multiprotein complex with ubiquitin ligase activity. Within this complex, pVHL is the subunit that recognizes protein substrates, stimulating their ubiquitination and proteasome-dependent degradation. The main target of this complex is the transcription factor hypoxia-inducible factor 1 (HIF-1*α*), which plays a key role in the cellular response to hypoxic conditions. It stimulates the transcription of genes involved in promoting angiogenesis and invasive growth. In renal cancer cells, this complex does not function; therefore, HIF-1*α* accumulates in cells and activates a cascade of other genes that encode factors that induce hypoxia, including VEGF or those involved in alternative pathways to VEGF, such as fibroblast growth factor receptor (FGFR), platelet-derived growth factor receptors (PDGFRs), AXL, and c-MET, all of which are involved in angiogenesis, tumor growth, and survival (14).

Zinc-finger and homeobox protein 2 (ZHX2) is a VHL target. VHL loss-of-function mutations usually result in an increased abundance and nuclear localization of ZHX2. Loss of ZHX2 inhibits signaling through the transcription factor NF-*κ*B, and ZHX2 binds to many NF-*κ*B target genes, revealing that ZHX2 is a potential therapeutic target for RCC (15).

VHL inactivation alone is insufficient for RCC tumorigenesis, and several gene mutations contribute to tumor heterogeneity that characterizes RCC. Intratumoral heterogeneity, defined as the presence of genetically different clones in different subpopulations of the same tumor, is a typical renal tumor condition (16). Accordingly, phylogenetic studies show how the tumorigenesis in the RCC follows an evolutionary model, ‘tree-like’: in the trunk lies the main mutation (*e.g.* the *VHL* gene in the clear cell tumor) that paves the way for tumorigenesis, and from the trunk, different subclonal mutations branch out, which contribute to tumor growth and progression. Data from the TRACERx renal study have identified secondary mutations and chromosomal changes involved in tumor evolution (17).

**Figure 1:**
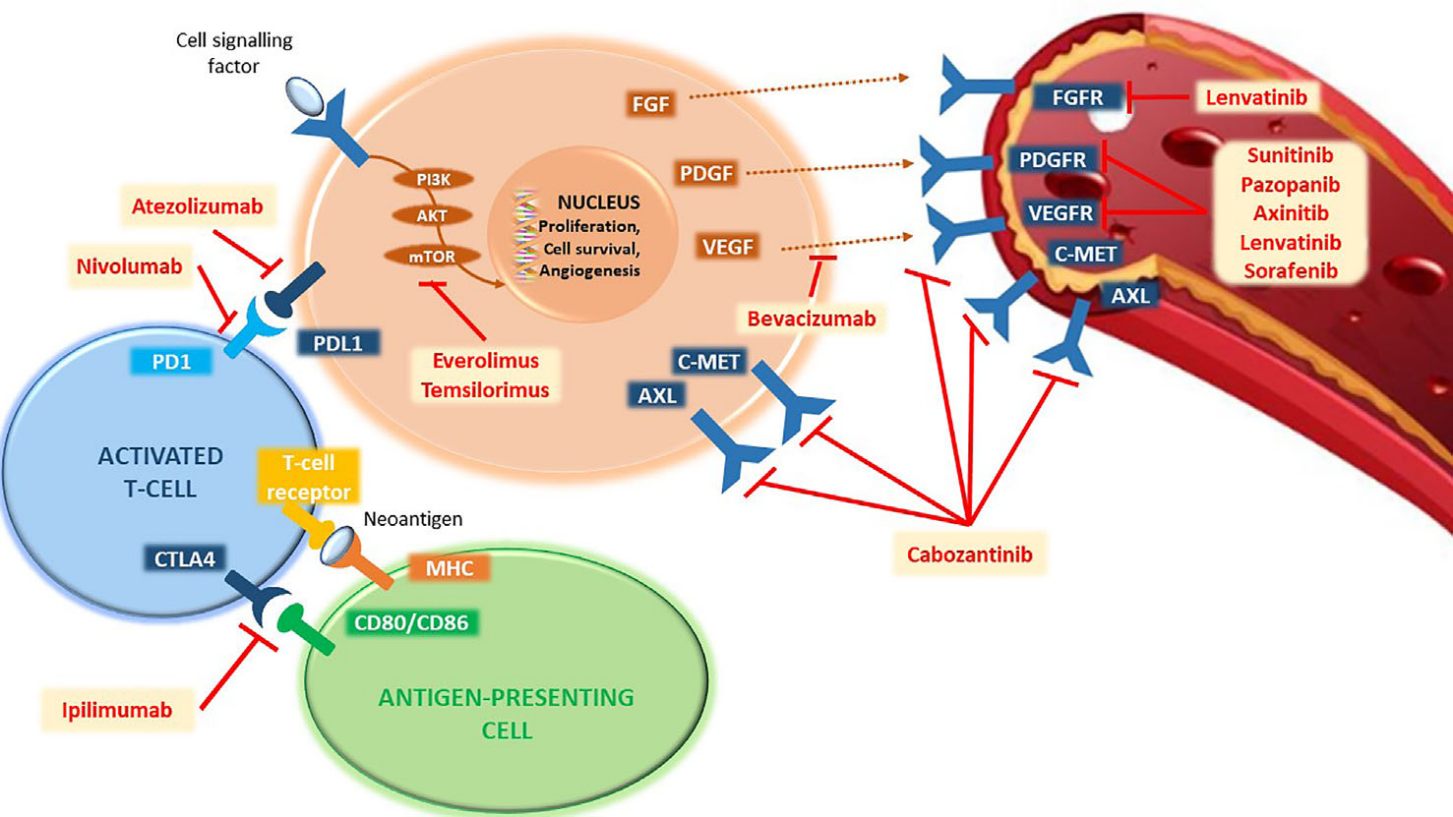
Representation of the main pathways involved in the mechanisms of tumorigenesis and proliferation of renal cancer cells and their targeted agents. PD1, programmed cell death-1 receptor; PD-L1, programmed death-ligand 1; CTLA4, cytotoxic T-lymphocyte-associated protein 4; CD80, cluster of differentiation 80; CD86, cluster of differentiation 86; MHC, major histocompatibility complex; PI3K, phosphatidylinositol-3-kinase; AKT, serine/threonine kinase 1; mTOR, mechanistic target of rapamycin; FGF, fibroblast growth factor; PDGF, platelet-derived growth factor; VEGF, vascular endothelial growth factor; cMET, mesenchymal epithelial transition factor; AXL, AXL receptor tyrosine kinase; FGFR, fibroblast growth factor receptor; PDGFR, platelet-derived growth factor receptor; VEGFR, vascular endothelial growth factor receptor.

Excluding hereditary forms, which cover only 4% of cases, for sporadic forms, The Cancer Genome Atlas (TCGA) has identified 19 genes involved in addition to *VHL*, including *BAP1, PBRM1, SETD2*, *KDM5C, KDM6A, mTOR, PTEN, PIK3CA*, and *p53* (18). The constitutive activation of the mTOR cascade plays an equally important role in renal tumorigenesis through the loss of p53 expression or mutation of genes such as *PI3K* and *PTEN*. Therefore, TKI therapies directed against one or more of these factors will always be a therapeutic weapon of fundamental importance, as these are precisely targeted against the genetic mechanisms based on tumorigenesis and proliferation of renal cancer cells (**Figure 1**).

In addition to proper genetic damage, we must consider the variations induced by the environment (epigenetics), alterations in receptor expression, and all the complexity that revolves around the tumor microenvironment. 

Systemic inflammation is frequently observed in advanced RCC (19). Nevertheless, the functional correlation between inflammation and RCC metastasis remains unclear. Recent data have demonstrated that cancer cells can secrete cytokines and chemokines through a process known as cancer-cell-intrinsic inflammation, altering the immune landscape (20–22). Cancer-cell-intrinsic inflammation contributes to cancer metastasis and the initial progression of cancers. The driver gene mutations responsible for the inflammation in different tumors are *TP53* and *KRAS* mutations (23–26). These mutations lead to increased cytokine release, which recruits myeloid cells in the primary tumor microenvironment or (pre-) metastatic sites.

It has been demonstrated that epigenetic remodeling determines the massive expression of inflammation-related genes in RCC. Synchronous inhibition of the bromodomain and extra-terminal motif suppressed C-X-C-type chemokines in clear cell RCC cells and decreased neutrophil-dependent lung metastasis, suggesting a potential therapeutic strategy (27).

The cells of the immune system (T cells, B cells, and natural killer cells), which represent the targets of known ICIs, such as anti-CTLA4, anti-PD-1, or anti-PD-L1, are found within the tumor microenvironment. In addition to playing a key role in the carcinogenesis process, some parameters such as the expression of PD-L1 have been associated with a worse prognosis (28) as well as a higher degree of tumor aggressiveness (29). Thus, the use of ICIs that block PD-1/PD-L1 binding or amplify the overall immune response finds in this biological rationale its high activity in patients with mRCC (**Figure 1**).

It remains evident that the intratumoral heterogeneity problem is responsible for the difficulty in identifying a single driver mutation and for overcoming mechanisms of clonal selection during targeted treatment (30). To make things worse, a microenvironment response exists: tumors treated with anti-angiogenic agents present an inflammatory infiltrate consisting mainly of regulatory T cells (CD4^+^FOXP3^+^) and express high levels of PD-L1, thus demonstrating the conditions associated with a worse prognosis (31). These findings suggest that the immunosuppressive phenotype found in metastatic sites, for example, is the result of close communication between the occurrence of anti-angiogenic treatment-resistant subclones and the enrichment of inflammatory infiltration with Treg cells to evade the anti-tumor immune response. Given the above-mentioned data, the rationale for combining TKIs with ICIs has become increasingly clear.

## The Latest Approved Therapeutic Strategies in mRCC

### Cabozantinib

Cabozantinib is a multi-targeting TKI directed against the receptors of factors involved in tumor growth, angiogenesis, pathological bone remodeling, chemoresistance, and metastatic progression of cancer, such as VEGF, MET, GAS6(AXL), RET, ROS1, TYRO3, MER, KIT (stem cell factor), TRKB, Fms-like tyrosine kinase-3 (FLT3), and TIE-2 (32). Based on its broad mechanism of action, it is believed to overcome resistance to anti-VEGF agents, such as sunitinib and pazopanib; thus, it was first tested as a second-line therapy in patients previously treated with anti-VEGF therapy (5) and subsequently as first-line therapy in patients with intermediate–poor-risk prognosis (6).

In the phase III METEOR trial, 658 patients with mRCC, who had previously been treated with at least one VEGF tyrosine kinase receptor inhibitor (VEGFR-TKI), were randomized 1:1 to receive cabozantinib (n = 330) or everolimus (n = 328), including those who may have previously been treated with other therapies, including cytokines and antibodies directed against VEGF, the PD-1 receptor, or other ligands. Additionally, patients with treated brain metastases were included. The primary endpoint of the study was progression-free survival (PFS). Secondary endpoints were objective response rate (ORR) and overall survival (OS). Most patients were males (75%), with a median age of 62 years. Seventy-one percent of patients had previously been treated with only one VEGFR-TKI. In 41% of patients, sunitinib was the single VEGFR-TKI previously received. According to the MSCKK criteria for the prognostic risk category, in 46% of patients, the prognosis was favorable; in 42%, it was intermediate (one risk factor); and in 13%, it was poor (two or three risk factors). In 54% of patients, three or more organs, including the lungs (63%), lymph nodes (62%), liver (29%), and bones (22%), had metastatic disease. The median duration of treatment was 7.6 months (range 0.3–20.5) for patients who received cabozantinib and 4.4 months (range 0.2–18.9) for patients who received everolimus. A statistically significant improvement has been demonstrated in PFS for cabozantinib compared to everolimus (7.4 months compared to 3.9 months, hazard ratio [HR] = 0.51 [0.41–0.62], p = 0.0001). In a subsequent interim analysis, a statistically significant improvement was also demonstrated in terms of OS [320 events, median value of 21.4 months compared to 16.5 months; HR = 0.66 (0.53, 0.83), p = 0.0003]. Comparable OS results were observed with a follow-up analysis (descriptive) at 430 events. Exploratory analyses of PFS and OS in the intent-to-treat population also showed consistent results in favor of cabozantinib compared to everolimus in different subgroups defined by age (<65 years compared to ≥65 years), sex, risk group, ECOG status (0 compared to 1), time from diagnosis to randomisation (<1 year compared to ≥1 year), tumor expression of MET (high compared to low compared to unknown), bone metastasis, visceral metastasis, number of VEGFR-TKIs previously received (one *vs* two), and duration of first treatment with VEGFR-TKI (≤6 months *vs >*6 months). Dose reductions were more frequent with cabozantinib than with everolimus, but no statistically significant difference in terms of discontinuation of severe adverse events was reported (5, 33).

The safety and efficacy of the first-line cabozantinib were evaluated in the CABOSUN study, a randomized, open-label, controlled *vs* sunitinib phase II study, which enrolled 157 mRCC patients, classified as intermediate or poor risk according to IMDC criteria. The patients (n = 157) were randomized 1:1 to receive cabozantinib (n = 79) or sunitinib with a schedule of 4 weeks on/2 weeks off (n = 78). The patients were stratified according to the IMDC risk category (81% intermediate and 19% poor) and the presence or absence of bone metastases. Approximately 75% of patients underwent nephrectomy before the start of treatment. The primary endpoint was the PFS, and the secondary endpoints were ORR and OS. Most patients were males (78%) with a median age of 62 years. Most patients (87%) had an ECOG performance status of 0 or 1; 13% had an ECOG performance status of 2. Thirty-six percent of the patients had bone metastases. The study has reached the primary endpoint of statistically significant improvement of the PFS for cabozantinib compared to sunitinib [8.6 months regarding 5.3 months; HR = 0.48 (0.32–0.73), p = 0.0005]. Patients showed a favorable effect with cabozantinib compared to sunitinib irrespective of MET status (positive or negative); however, cabozantinib demonstrated greater activity in patients with positive MET status than that in patients with negative MET status [HR = 0.32 (0.16 and 0.63) *vs* 0.67 (0.37 and 1.23)]. In addition, compared to the treatment with sunitinib, treatment with cabozantinib has been associated with a trend of longer OS (30.3 months compared to 21.0 months; HR 0.74 [0.47–1.14]) (6).

In the two aforementioned studies, the most frequently reported serious adverse events with cabozantinib were hypocalcemia, hypokalemia, thrombocytopenia, hypertension, palm-plantar erythrodysesthesia syndrome, proteinuria, and gastrointestinal events (abdominal pain, inflammation of the mucous membranes, constipation, diarrhea, and vomiting) and were generally found during the first 8 weeks of treatment. In the METEOR study, dosing reductions and dosing interruptions of 59.8 and 70%, respectively, occurred in relation to an adverse event caused by cabozantinib. In CABOSUN, where patients were naïve to treatment, the percentages of reduction and treatment interruption were quite similar (46 and 73% of patients, respectively). Therefore, it does not seem to be a condition of drug toxicity. However, hypertension has been observed more frequently in the population of naïve patients (67%) than in patients included in the METEOR trial who had been previously treated with anti-VEGF targets (37%).

### Nivolumab: Monotherapy and ICI Combination Therapy

Nivolumab was the first anti-PD-1 ICI approved for the treatment of mRCC, first as monotherapy in patients previously exposed to a VEGFR-TKI and then in combination with ipilimumab as the first-line treatment in patients with intermediate- and poor-risk prognosis. According to data from the Phase III Checkmate 025, patients who progressed during or after 1–2 previous anti-angiogenic regimens were eligible for treatment with nivolumab monotherapy (34). This study included patients regardless of tumor PD-L1 status and with a 70% Karnofsky performance status (KPS). Patients with a history of brain metastasis or concomitant brain metastasis, previously treated with an mTOR inhibitor, affected with an autoimmune disease in the active phase, or with medical conditions requiring systemic immunosuppression were excluded from the study. A total of 821 patients were randomized to receive nivolumab (n = 410) or everolimus (n = 411). The study reached the primary endpoint of efficacy (median OS equal to 25 months with nivolumab compared to 19.6 months with everolimus, HR = 0.73 [0.7–0.93], p = 0.0018). Secondary endpoints included ORR and PFS, as evaluated by the investigator. In this study, nivolumab was shown to be better than everolimus in pre-treated patients in terms of ORR (25 *vs* 5%, p < 0.001, HR for OS = 0.73; 95% confidence interval (CI) = 0.57–0.93). However, no significant advantages in terms of PFS have been reported.

Nivolumab in combination with ipilimumab proved to be superior to sunitinib as the first-line therapy in the Phase III study Checkmate 214 (8). The study included patients with mRCC, with clear cell components that were not previously treated. The primary efficacy population included patients at intermediate/poor-risk according to the IMDC criteria. A total of 1,096 patients were enrolled, of which 847 at intermediate/poor-risk were randomized to nivolumab in combination with ipilimumab (n = 425) for four cycles followed by nivolumab monotherapy or sunitinib (n = 422). The primary endpoints were the OS, ORR, and PFS. Patients with mRCC with intermediate/poor prognosis according to IMDC reported a statistically significant benefit in terms of both OS and ORR (HR for OS = 0.63, 95% CI = 0.44–0.89; ORR 42 *vs* 27%, p < 0.001), regardless of the expression level of PD-L1, although in the PD-L1 >1% group, the advantage was even more significant (HR = 0.52; 95% CI = 0.34–0.78). The PFS was not significantly different between the two groups (HR = 0.82; 95% CI = 0.64–1.05). In addition, in the 249 patients at favorable risk, nivolumab plus ipilimumab was detrimental in terms of OS compared to sunitinib (HR = 1.13 [0.64–1.99] p = 0.6710). In terms of tolerability, the combination of ipilimumab and nivolumab was burdened with a higher toxicity than sunitinib (22 *vs* 12% of patients, respectively, discontinued treatment for toxicity) (8) and compared to IO with a single agent, resulting in a more severe immune-related toxicity percentage (35). However, a more recent report on the Checkmate 214 study demonstrated that patient-reported outcomes were more favorable with nivolumab plus ipilimumab than sunitinib in patients at intermediate or poor risk, leading to fewer symptoms and better health-related quality of life (36). Moreover, to better characterize the association between outcomes and IMDC risk in CheckMate 214, a *post-hoc* analysis (n = 1051) of efficacy by the number of IMDC risk factors was completed. ORR with nivolumab plus ipilimumab was consistent across zero to six IMDC risk factors, whereas with sunitinib, it decreased with an increasing number of risk factors. The benefits of nivolumab plus ipilimumab over sunitinib in terms of ORR (40–44% *vs* 16–38%), OS (HR = 0.50–0.72), and PFS (HR = 0.44–0.86) were consistently observed in subgroups with one, two, three, or four to six IMDC risk factors. These results demonstrate the benefit of first-line nivolumab plus ipilimumab over sunitinib across all intermediate- and poor-risk groups, regardless of the number of IMDC risk factors (37).

Thanks to the data reported, the combination of nivolumab and ipilimumab was approved by ESMO guidelines in intermediate- and poor-risk prognostic subgroups of mRCC.

Moreover, a *post-hoc* analysis of nivolumab plus ipilimumab or sunitinib in IMDC intermediate/poor-risk patients with previously untreated mRCC with sarcomatoid features showed an ORR of 56.7% (CI = 43.2–69.4, p < 0.001) in the combination arm against 19.2% (9.6–32.5) of standard treatment and a rate of complete response (CR) of 18.3% in the experimental group, whereas no CR was observed in the sunitinib arm (38).

Elderly patients with pre-treated mRCC may benefit from therapy with nivolumab or nivolumab plus ipilimumab as a first-line option (7, 39), and salvage-line cabozantinib may offer the best survival outcomes, although evidence suggests that the majority of first-line treatments have worse efficacy in older patients than in younger patients (40, 41).

Despite the undeniable benefits of ICIs in the treatment of mRCC, some aspects must be considered: i) only a subset of patients achieves objective responses, ii) some patients have a delayed response, and iii) a significant number of patients do not benefit even clinically. In detail, although the so-called ‘combo’ IO is particularly active as the upfront treatment in patients with intermediate/poor prognosis, it cannot be a universal choice for all patients, but only for those patients ‘fit’ for a more intensive combined treatment. Moreover, the ipilimumab–nivolumab combination was less effective than sunitinib in patients over 75 years of age, who represent most of those we met in clinical practice. Therefore, IO is an important strategy both as first- and second-line treatment in patients with mRCC, but TKI agents remain the central focus of mRCC treatment in all therapeutic lines. Several hypotheses have been formulated regarding the lack of efficacy of ICIs in all patients, and among these, tumor heterogeneity and the dynamism of the tumor microenvironment typical of renal cancer cells seem to be the main conditions (29, 42).

### The Combination of VEGF-Targeting Agents With ICIs

The upfront combination of VEGF-targeting agents with ICIs is emerging as a therapeutic alternative that could overcome the limitations of IO alone as well as target both the cascade of angiogenesis and the tumor microenvironment (**Figure 1**). Anti-VEGFR inhibitors, in addition to their intrinsic anti-angiogenic effect, showed immunomodulatory effects: unlocking the inhibitory brake of VEGF, promoting infiltration and activation of effector cells, and inhibiting immunosuppressive cells (43). Although the initial studies of sunitinib or pazopanib associated with nivolumab had negative results for the high rates of liver and gastrointestinal toxicity (44), new combinations are proving to be active and well tolerated (45–47).

In the IMmotion151 study, the anti-PD-L1 atezolizumab combined with the anti-VEGF bevacizumab performed better than sunitinib monotherapy in patients with PD-L1-positive tumors (HR = 0.74 [95% CI = 0.57–0.96]; p = 0.02]; however, in the intention-to-treat (ITT) population, the median OS was 33.6 months in the combination arm *vs* 34.9 months in the sunitinib arm, and the results (HR = 0.93) had not yet crossed the significance boundary (45). A pre-specified subgroup analysis of IMmotion151 demonstrated a significant benefit in terms of PFS in patients with mRCC with sarcomatoid features in the bevacizumab plus atezolizumab treatment arm when compared with the sunitinib treatment arm (48).

Other promising combinations always used as first-line treatment are axitinib plus avelumab and axitinib plus pembrolizumab, tested in the Javelin Renal 101 (46) and the Keynote-426 (47) trials, respectively.

The Javelin Renal 101 randomized 442 and 444 patients to the avelumab plus axitinib and sunitinib arms, respectively, and showed that the combination treatment was higher than sunitinib monotherapy in terms of PFS and ORR, regardless of the PD-L1 status and prognostic risk category (46). The last update of the study confirmed previous results; in particular, in the overall population, the median PFS was 13.3 (95% CI = 11.1e15.3) *vs* 8.0 months (95% CI = 6.7e9.8), HR = 0.69 [95% CI = 0.574–0.825]; p < 0.0001); moreover, the combination prolonged PFS2 compared with sunitinib. However, OS data (primary endpoint of both studies) are still immature (49).

The Keynote 426 study is a phase 3 trial that randomly assigned 861 patients with previously untreated advanced RCC to receive pembrolizumab plus axitinib or sunitinib. The primary endpoints were the OS and PFS in the ITT population. The key secondary endpoint was ORR. After a median follow-up of 12.8 months, this study observed a significant benefit in terms of PFS (15.1 *vs* 11.1%, HR = 0.69; 95% CI = 0.57–0.84; p = 0.001) and ORR (59.3 *vs* 35.7%, p = 0.001) in favor of the combined treatment arm, disregarding the status of PD-L1 and the prognostic risk category (47).

The results of the extended follow-up of the randomized phase III study KEYNOTE-426 (median follow-up 30.6 months) confirmed the benefit for the experimental arm, which was proven statistically significant in terms of median OS [not reached with pembrolizumab and axitinib *vs* 35.7 months (95% CI = 33.3–not reached) with sunitinib; HR = 0.68 (95% CI = 0.55–0.85), p = 0.0003], median PFS [15.4 months with pembrolizumab and axitinib (12.7–18.9) *vs* 11.1 months for sunitinib (95% CI = 9.1–12.5); HR = 0.71 (95% CI = 0.60–0.84), p = 0.0001], and ORR (60% in the combo arm *vs* 40% in the sunitinib group). Although the trial was not designed to observe differences between risk categories, it should be noted that the benefit in terms of OS was particularly evident in the population at intermediate and unfavorable risk [pembrolizumab plus axitinib *vs* sunitinib: HR = 0.63 (95% CI = 0.50–0.81)], while it was not significant in the favorable risk group [HR = 1.06; (95% CI = 0.60–1.86)]. Moreover, in terms of toxicity, no significant news emerged with the continued follow-up of patients in the study. The most frequent treatment-related grade 3 or higher adverse events (10% of patients in both groups) were hypertension [95 (22%) of 429 patients in the pembrolizumab group plus axitinib *vs* 84 (20%) of 425 patients in the sunitinib group], increased alanine aminotransferase levels [54 (13%) *vs* 11 (3%)], and diarrhea [46 (11%) *vs* 23 (5%)] (50).

The fact that the advantage in OS for the combination, already known from the first analysis, is maintained over time, although half of the patients randomized to only sunitinib had then received progression IO (*vs*. 8% in the experimental arm), suggests the synergistic activity of the combination of pembrolizumab plus axitinib, which may therefore not be reproducible by their use in sequence. With regard to drug synergy, the role of a single agent in the overall result may also be different: while axitinib is more responsible for shrinkage, pembrolizumab could then be more decisive in maintaining the volumetric reduction effect over time (51).

Furthermore, although all the front-line combination trials enrolled patients with clear cell RCC, exploratory *post-hoc* analyses from these studies demonstrated that patients with sarcomatoid differentiation, which has historically been associated with worse prognosis, derive marked benefits from ICI-based therapy. Based on these data, the Food and Drug Administration (FDA) and EMA in 2019 approved the axitinib–pembrolizumab combination as the first-line treatment for patients with clear cell mRCC in the all-risk category.

CheckMate-9ER is a randomized controlled trial comparing the combination of nivolumab and cabozantinib *vs* sunitinib as a first-line treatment for mRCC with a clear cell component and any IMDC risk group. In the first analysis of the study, the superiority of the combination arm over standard treatment was shown to meet all three efficacy endpoints, with a 40% reduced risk of death [HR = 0.60 (98.89% CI = 0.40–0.89); p = 0.0010; median OS not reached in both arms]. In patients treated with the combination cabozantinib and nivolumab, the median PFS, the primary endpoint of the study, is doubled compared to patients who received only sunitinib: 16.6 months compared to 8.3 months [HR = 0.51 (95% CI = 0.41–0.64), p = 0.0001]. In addition, cabozantinib in combination with nivolumab showed a higher ORR (56 *vs* 27%) and 8% of patients compared to 5% who achieved a complete response. Moreover, the combination treatment was associated with a longer response duration compared to sunitinib, with a median duration of 20.2 months compared to 11.5 months. In addition, patients treated with the combination showed a lower rate of discontinuation of treatment than those treated with sunitinib (44.4 *vs* 71.3%) and a significantly lower rate of discontinuation for disease progression than sunitinib (27.8 *vs* 48.1%). All these key efficacy results are consistent in pre-specified subgroups and all risk categories according to the IMDC and PD-L1 expression (52, 53). Based on this study, the ESMO guidelines proposed the combination of nivolumab and cabozantinib as a valid first-line therapy in all prognostic subgroups (52).

Unlike the KEYNOTE 426 trial, no significant results were obtained in terms of OS when the experimental arm was compared with the standard treatment in the other phase III studies. In fact, in the Javelin Renal 101 study, OS data were immature in the 2019 publication, while in the IMmotion-151 trial, OS was not met.

Among the new drug combinations tested in mRCC, there is also the one examined in the phase Ib/II TiNivo study, which evaluated the efficacy and safety of combination therapy with nivolumab plus tivozanib, a highly potent and selective VEGFR-TKI approved by the European Medicines Agency (EMA) for first-line treatment of patients with mRCC (54), and showed a generally tolerable profile and promising anti-tumor efficacy (55).

## How Therapeutic Algorithm Has Changed in mRCC Treatment With the Approval of Combo?

For a decade, it has been wondered what the best sequence treatment between TKI–mTORi–TKI *vs* TKI–TKI–mTORi is. However, the next future question will be much more complex since there are no comparative studies, clear prognostic factors, or predictive markers, thus making a weighted choice between the various options available in the first- and second-line very difficult.

The new treatment strategies range from molecular targeted agents such as cabozantinib, able to overcome some anti-angiogenic mechanisms of resistance, through ICIs, such as nivolumab, as a single agent, up to the combinations of ICIs (nivolumab + ipilimumab), or between ICIs with VEGF-targeting agents (atezolizumab + bevacizumab, pembrolizumab + axitinib, avelumab + axitinib, cabozantinib + nivolumab, and others under investigation).

The paradigm of first-line treatment in advanced RCC, firmly occupied for more than 10 years by monotherapy with anti-angiogenic TKIs, such as sunitinib or pazopanib, has changed, and combinations of ICIs, either with each other or with TKIs, have shown efficacy compared to monotherapy with TKIs. In light of the results of recent combinations, except for comorbidity and clinical contraindications, in the first-line, the therapeutic proposal is to administer all prognostic classes the combination TKI/IO (axitinib plus pembrolizumab/nivolumab plus cabozantinib) or IO/IO (nivolumab plus ipilimumab), and considering the combination IO/IO for patients with sarcomatoid components, whereas all other cases remain valid for TKI monotherapy, in particular, cabozantinib in the intermediate- and high-risk subgroups unfit for combo treatment, and pazopanib or sunitinib in the good risk unfit for combo (56).

Whether the objective is to achieve a complete response (CR) as well as a long survival (or possible cure), ipilimumab plus nivolumab or nivolumab plus cabozantinib would be the treatment of choice. In fact, the CR rates in CheckMate 214, CheckMate9ER, Keynote426, and Javeline Renal 101 were 9, 8, 5.8, and 3.8%, respectively. In contrast, we should also consider that a higher rate of progressive disease (PD) was observed as the best response to treatment in the CheckMate 214 trial, while the lowest was observed in CheckMate9ER. The toxicity profile is a further discriminant in the choice of combination treatment. In fact, for the IO–IO combination, the major toxicities are limited to the induction phase with ipilimumab, while for the combination of an ICI and a VEGFR-TKI, safety issues tend to persist over time due to the prolonged administration of both agents.

As the field stands now, the immuno-target combination could represent a particularly valid opportunity, especially in patients with a ‘cold’ phenotype, whose tumor is characterized by poor immune infiltration and are considered less likely to respond to ICI-based treatment alone.

Given the lack of head-to-head comparative studies, both experience and common sense must guide the choice of a physician according to the following considerations: i) patient characteristics, comorbidities, drug interactions with concurrent therapy, occupation, preferences of patient, and side effects that can affect the quality of life; ii) neoplasia features, its histology, if it has a representative sarcomatoid components, the genetic structure, the burden of cancer disease, and the location of the metastases and their related symptoms; iii) balancing the risk and benefit of treatment itself: for safety, we should consider that the trade-off between efficacy and safety that a first-line patient is willing to accept is usually unbalanced in favor of efficacy; iv) biological aggressiveness of the tumor: in the case of an aggressive disease, the combo IO/TKI seems a very reasonable choice to control disease growth while waiting for the tail effect of IO; otherwise, one could head for the long-term benefit of the IO/IO combo, as well as for complete response, trying to spare the additional toxicity derived from the continuous use of the VEGFR-TKI.

Moreover, a recent meta-analysis network on the choice of the first-line showed that cabozantinib is the best molecular targeted agent for the advantage in terms of PFS in patients at intermediate/poor risk compared to sunitinib, with a 91% chance of giving the best benefit in PFS (57). Therefore, the choice will be conditioned by our primary endpoints, even if they do not always coincide with those of large clinical trials.

**Figure 2:**
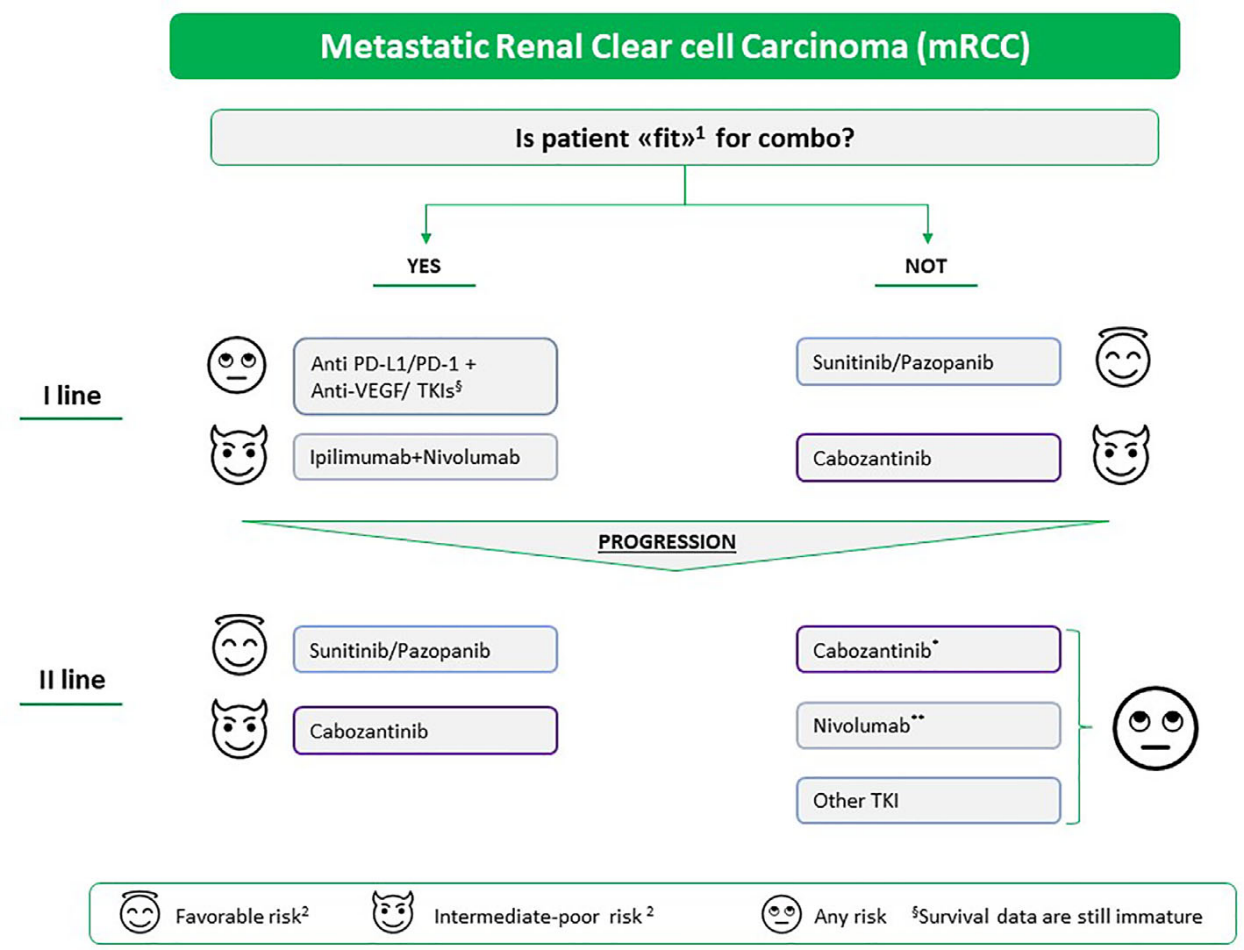
Proposed therapeutic algorithm for the treatment of mRCC in and beyond the first-line setting. The choice of treatment is based on (1) i) patient characteristics: comorbidities, potential drug interactions with the concomitant therapy, occupation, patient preferences and the side effects that can affect the quality of life; ii) and tumor characteristics: histology, if it has a representative sarcomatoid component, the genetic structure, the tumor burden, site of metastases, and related symptoms. (2) MSKCC/IMDC prognostic classification; *if not previously carried out, **if the patient does not have autoimmune disease in the active phase, solid organ transplant, or interstitial pneumopathy or if the patient requires high doses of corticosteroids. TKI, tyrosine kinase inhibitor Pazopanib and cabozantinib are still not indicated as second-line treatment after immunotherapy; however, real-world analysis of patients treated with cabozantinib after anti-PD-1 treatment reported promising results.

Taking into account that the IMDC prognostic model was developed at the time of a first-line anti-VEGF-based therapy (58) and that neither validated prognostic models in first-line with ICIs or with the immuno-target combo nor data on the second-lines are available, the therapeutic algorithm of mRCC could be revised in the following way (**Figure 2**): i) for the first-line, to assess whether the patient is considered ‘fit’ for a combination strategy; ii) for subsequent lines, taking into account what has been done previously (in immuno-naive patients, the choice could fall on nivolumab or another TKI such as cabozantinib, while in TKI-naive patients, the choice could fall on an anti-VEGF TKI such as sunitinib, pazopanib, or cabozantinib). Nevertheless, data on pazopanib or cabozantinib as second-line treatment after ICI-based treatment are not available. However, cabozantinib demonstrated impressive PFS and OS when administered post-IO in patients with mRCC, according to findings from the METEOR sub-analysis^33^ and recent retrospective real-world studies (59, 60). After disease progression to first-line TKI-based monotherapy, the factors that could guide the choice towards a second-line of treatment in favor of another TKI are low or intermediate risk, long duration of first-line treatment with VEGFR-TKI, good tolerability to previous treatment lines, low tumor burden, slow progression, revascularization of pre-existing lesions, and high probability of receiving further treatment lines. In favor of IO-based second-line treatment, we have considered the following factors: high risk, short duration of first-line treatment with VEGFR-TKI, poor tolerability to TKI, dose reductions and interruptions, high tumor burden, rapid progression, progression not guided by angiogenesis, and low probability of receiving further therapy.

Currently, there are no data comparing the available strategies that combine two IO agents or TKI plus IO, but increasing evidence suggests that some biomarkers and genetic features could guide optimal treatment options for patients.

## What to Expect From Diagnostic Imaging?

The complex therapeutic scenario described above makes imaging evaluation extremely challenging, both at the time of diagnosis and in assessing the response to treatment. At the time of diagnosis, owing to high intratumoral heterogeneity and heterogeneity between the gene expression profiles of primary cancer and its metastases, tumor genomic characterization is necessary. Considering the technical difficulties and morbidity in performing multiple renal biopsies (61), a solution may be represented by radiogenomics. Radiogenomics, a result of advances in both computational hardware and machine-learning algorithms, is an emerging field in which quantitative information is extracted from radiological images (radiomics) and is correlated with tumor genomic profiling (62). Although studies are still preliminary (63, 64), it is expected that quantitative imaging data might become a useful biomarker for assessing tumor prognosis, treatment selection, and prediction of treatment response.

With the advent of anti-VEGF and TKIs and then ICIs, the evaluation of response to therapy made it necessary to introduce new objective response criteria [*i.e.* modified Choi (65), SACT (66), iRECIST (67)], since conventional RECIST (66) is not adequate for categorizing patient response. However, there are still open issues regarding the assessment of pseudo-progression and dissociated response (68, 69), both of which are strongly associated with the clinical benefit of ICIs and hyper-progression. Further challenges will await radiology with the advent of combined treatments. The solution will probably be found in the integrated analysis of imaging data (from different sources, including CT, MRI, and PET, combining morphological and functional studies, targeting tumor perfusion and cellularity), tumor mutational burden, and biological markers. Once collected, this large amount of data will be processed by high-speed processors driven by artificial intelligence.

## Possible Future Predictive and Prognostic Biomarkers

### PD-L1 Expression

Several studies have demonstrated the negative prognostic role of the expression of PD-L1 in the setting of mRCC (70–72). The expression of PD-L1 on tumor cells was associated with a higher tumor stage and a worse response to TKI therapy in two *post-hoc* analyses of the COMPARZ study and the METEOR and CABOSUN trials (28, 72–74). In addition, a meta-analysis including more than 1,300 patients showed that higher PD-L1 expression correlated with an approximately doubling risk of death (75). In contrast, the predictive role of PD-L1 in response to IO is still controversial, and the results obtained in the exploratory analyses of clinical trials investigating ICIs are inconclusive (7, 8, 45–47). In the CheckMate 025 trial, PD-L1 expression was associated with poor survival independent of the treatment received, but not with response to nivolumab (7). The CheckMate 214 trial showed a higher PFS in the ipilimumab plus nivolumab arm than in the sunitinib arm for IMDC intermediate–poor-risk patients, with PD-L1 expression in 1% or greater of cells (median PFS 22.8 *vs* 5.9 months), but this advantage was not observed when PD-L1 was less than 1% (median PFS, 11 *vs* 10.4 months). Conversely, a better ORR and OS for IO over an anti-vascular agent was reported regardless of tumor PD-L1 expression level (8). In the IMmotion 151 trial, the magnitude of benefit derived from the combination therapy with atezolizumab plus bevacizumab increased in patients with PD-L1 expression by more than 1% of tumor-infiltrating lymphocytes compared with the ITT population (45). In the JAVELIN Renal 101 and KEYNOTE-426 trials, the combination therapy showed a benefit over sunitinib irrespective of PD-L1 expression (49, 50).

The above-mentioned results suggest that the expression of PD-L1 in mRCC cannot completely predict the responsiveness of tumors to ICIs. Its role remains controversial and warrants further investigation. Moreover, the assessment method and tumor heterogeneity are the major limitations of the evaluation of PD-L1 (76). The technique used for the IHC analysis has an elevated variability among the different methods available, and the scoring systems are not concordant for the target cells evaluated, whether tumor cells, immune cells infiltrating the neoplastic stroma, or a combination of both; additionally, there is no validated positivity cut-off (77, 78). Furthermore, PD-1 and PD-L2 evaluation should be considered, and their role should be clarified (79). Finally, the expression of PD-L1 is dynamic, changing depending on the history of the disease and the treatments received. In addition, intratumoral variability and a different expression in the primitive tumor and metastases, which would explain the high response rates obtained despite the negativity of PD-L1 in the primitive lesion, should be considered when the expression of this biomarker is examined (80).

### Tumor Mutational Burden

TMB is defined as the total number of mutations per coding area of the tumor genome, measured as mutations per megabase (mutations/Mb) (81). In tumors with high TMB, there is an increased production of surface neoantigens that stimulate the anti-tumor immune system response, which could explain the potential association between TMB and response to ICIs (82). In the setting of mRCC, TMB is variable, ranging from a very low level in chromophobe type to a higher value in clear cell and papillary tumors and is not concordant with the clinically defined prognostic groups according to IMDC and MSKCC (83). Regarding its prognostic role, the data in the literature are discordant, since some studies observed a correlation between improved survival and increased TMB, while others demonstrated a negative prognostic role (84, 85). Concerning the predictive significance of TMB, no association between TMB and survival, PD-L1 expression on tumor cells, or clinical benefit was observed (86).

### Microenvironment

RCC is characterized by a heterogeneous population of tumor-infiltrating immune cells; however, conflicting data have been obtained to date. Infiltration of effector T cells, such as CD8^+^ lymphocytes, and M1 macrophages may be associated with a better prognosis, whereas infiltration of regulatory T cells, such as Tregs and M2 macrophages, have a poorer outcome (87–90). In contrast, high intra- and peri-tumoral CD8^+^ cell density was also correlated with poor prognosis (91). It was demonstrated that PD-L1 expression on tumor cells could lead to higher CD8^+^ T cell infiltration, distinguishing two groups of tumors with CD8^+^ infiltrate, and the group with low expression of immune checkpoints and localization of mature dendritic cells was associated with a good prognosis (92).

Concerning the predictive role of the microenvironment, a comprehensive analysis of patients enrolled in four clinical trials on nivolumab demonstrated a poorer and greater response in correlation to the overexpression of genes involved in metabolic functions (*e.g. UGT1A*) and the increased expression of immune markers (*e.g.* BACH2 and CCL3), respectively (93). Moreover, an exploratory analysis of the IMmotion150 trial reported that a T-effector immune gene in association with the expression of PD-L1 and the infiltration of T CD8^+^ cells correlates with a higher ORR and prolonged PFS in the atezolizumab arm (45). In particular, it was observed that VEGF blockade could promote the infiltration of T cells into the tumor microenvironment, thus potentiating the mechanism of action of ICIs (94).

### Circulating Tumor Markers

Circulating tumor DNA (ctDNA) and circulating tumor cells (CTCs) are peripherally detectable tumor-derived materials. These markers could detect primary and metastatic sites non-invasively and evaluate the response to therapy (95–97). Variable frequencies of genomic alterations were detected in the front-line and second-line treatment settings, showing an increased incidence of genomic alterations, particularly those affecting TP53 and MTOR, after first-line treatment with VEGFR-TKI therapy (96). These differences could reflect treatment-selective pressures and the effect of front-line therapy on ctDNA load, but might also simply depend on the technical limitations of ctDNA assessment in this disease (98).

Other circulating protein and lipid markers have demonstrated predictive and prognostic value in advanced disease. Based on 52 circulating markers, a cohort of 69 patients treated with first-line sorafenib was grouped by either an angiogenic or an inflammatory signature, with correlations to PFS (HR = 0.2 *vs* 2.25; p = 0.0002) (99). Additional markers in serum have been investigated, such as soluble VEGF, circulating microRNAs, carbonic anhydrase 9, and inflammatory markers, such as IL-6 and IL-8, but most of these studies were conducted in the era of targeted therapies (99–103), and new dedicated investigations are required to address the dramatic changes in treatment paradigms brought about by the advent of ICIs.

### Genomic and Transcriptomic Environments

There are three possible treatment strategies in the therapeutic landscape of mRCC: angiogenic inhibitors at one end, IO at the other, and combinations of the two classes in the middle. The challenge, however, is identifying the subset of patients who could benefit from one therapeutic class alone to avoid the unnecessary toxicity of combination approaches.

Using RNA-based analyses, four distinct molecular subgroups associated with different responses and survival were defined: Cluster 3 had the best prognosis with high angiogenic gene expression and was associated with a better outcome under anti-angiogenic therapy (*PBRM1* mutation was frequently associated) (104); Cluster 4, with upregulation of the immune pathway, had a worse prognosis, with a frequent sarcomatoid differentiation and expression of PD-L1 (105); and Clusters 1 and 2 were intermediate clusters with a lower expression of angiogenic and immune genes. These results may have the potential to inform treatment personalization in patients with mRCC (106).

The phase 2 IMmotion150 trial investigated the efficacy, as measured by PFS, of atezolizumab with or without bevacizumab against sunitinib in patients with untreated mRCC and correlated differential gene expression signatures (angiogenesis, T-effector, and myeloid) with therapeutic response. Highly angiogenic tumors, which coincided with tumors exhibiting *PBRM1* mutations, seemed to benefit more from sunitinib, but not from atezolizumab either alone or in combination with bevacizumab. The combination treatment improved clinical benefits compared with sunitinib in T-effector high tumours. Tumors with T-effector high and lower myeloid inflammation-associated gene expression benefited from atezolizumab monotherapy. Instead, in T-effector high tumors, a concomitant high myeloid inflammation predicted a worse response to IO alone. Myeloid inflammation is associated with high expression of IL-6, prostaglandins, and the CXCL8 family of chemokines, which suppress the anti-tumor immunity. The improved clinical outcome associated with atezolizumab + bevacizumab compared with atezolizumab monotherapy in this subgroup suggests that the addition of bevacizumab to atezolizumab may overcome innate inflammation-mediated resistance in these tumors (104).

Based on the analysis of the angiogenic profile in comparison with the immunological profile of the study IMmotion151, it is possible to define subgroups of tumors that benefit from different treatment strategies. Given the new associations, it would be interesting to evaluate these aspects in other combinations of IO/TKI and see if, for example, the addition of TKI modifies the immunogenicity of these tumors.

## Conclusions

Considering the continuously evolving scenario in the treatment of patients with mRCC, the future goal will be to better characterize renal neoplasia in all its complexity, from the trunk to the last of its branches. However, to outline the most appropriate treatment path for each patient, we cannot deny that only clinical criteria are very likely to understand the needs of patients. Given the significant improvement in therapeutic options, prospective studies are needed that would elucidate: what will be the most effective therapeutic algorithm and how patients will be selected to hit more targets; will it be more effective to use therapeutic agents in sequence or by focusing completely on the first therapeutic line; will it be more effective to use a combination strategy from the beginning of mRCC treatment? Further studies are required to answer these questions.

## Author Contributions

All authors have read and agreed to the published version of the manuscript. All authors listed have made a substantial, direct, and intellectual contribution to the work and approved it for publication.

## Conflict of Interest

The authors declare that the research was conducted in the absence of any commercial or financial relationships that could be construed as a potential conflict of interest.
